# Novel Positron Emission Tomography Tracers for Imaging Vascular Inflammation

**DOI:** 10.1007/s11886-020-01372-4

**Published:** 2020-08-09

**Authors:** Andrej Ćorović, Christopher Wall, Justin C. Mason, James H. F. Rudd, Jason M. Tarkin

**Affiliations:** 1 Division of Cardiovascular Medicine, University of Cambridge, Cambridge, UK; 2 Cardiovascular Division, National Heart & Lung Institute, Imperial College London, London, UK

**Keywords:** PET, Inflammation, Atherosclerosis, Large-vessel vasculitis, Molecular imaging, Non-invasive imaging

## Abstract

**Purpose of Review:**

To provide a focused update on recent advances in positron emission tomography (PET) imaging in vascular inflammatory diseases and consider future directions in the field.

**Recent Findings:**

While PET imaging with ^18^F-fluorodeoxyglucose (FDG) can provide a useful marker of disease activity in several vascular inflammatory diseases, including atherosclerosis and large-vessel vasculitis, this tracer lacks inflammatory cell specificity and is not a practical solution for imaging the coronary vasculature because of avid background myocardial signal. To overcome these limitations, research is ongoing to identify novel PET tracers that can more accurately track individual components of vascular immune responses. Use of these novel PET tracers could lead to a better understanding of underlying disease mechanisms and help inform the identification and stratification of patients for newly emerging immune-modulatory therapies.

**Summary:**

Future research is needed to realise the true clinical translational value of PET imaging in vascular inflammatory diseases.

## Introduction

Inflammation is the cause or consequence of many cardiovascular diseases. In particular, inflammation is central to the pathogenesis of atherosclerosis [1], the most common cause of myocardial infarction and ischaemic stroke. Large-vessel vasculitis (LVV) is another important vascular inflammatory disease, which is associated with progressive arterial injury and accelerated coronary atherosclerosis.

Non-invasive imaging is a key component of the diagnostic and disease-monitoring pathways for these cardiovascular inflammatory diseases. While echocardiography, CT, MRI, and nuclear perfusion imaging are first-line non-invasive cardiac investigations, positron emission tomography (PET) imaging of inflammation can also play an important clinical role. Moreover, advances in cardiovascular PET imaging research and technology, including hybrid PET/MRI and total body PET, may open new clinical translational avenues in the near future.

In atherosclerosis, vascular inflammation detected by PET may serve as a marker of high-risk plaques or overall heightened disease activity. This approach could be particularly important in the post CANTOS (Canakinumab Anti-inflammatory Thrombosis Outcome Study) [2••] and COLCOT (Colchicine Cardiovascular Outcomes Trial) [3••] era, either as a means of testing drug efficacy in clinical trials or identifying and stratifying groups of individuals who would most benefit from novel anti-inflammatory therapies. Moreover, while PET imaging is already an established technique for diagnosis of LVV, further research is needed to evaluate its role for tracking therapy responses and identifying individuals with residual or refractory disease that require treatment escalation.

This article aims to provide a focused update on recent research relating to PET imaging of vascular inflammation in atherosclerosis and LVV. Specifically, we will highlight three key areas of ongoing research that may be the most clinically relevant in coming years:

i) Evaluation of novel PET tracers for imaging specific components of the vascular immune response
ii) Use of PET imaging in the clinical trials pipeline to gain early insights about drug efficacy
iii) Investigation of PET imaging for identification and stratification of high-risk patients for emerging immunomodulatory therapies.

## ^18^F-FDG PET Imaging of Vascular Inflammation

PET “tracers” are ligands or molecules of interest that are labelled with positron emitting radionuclides, allowing them to be localised in the body after injection by the detection of gamma rays, the by-product of annihilation events occurring when a positron encounters an electron. While PET is extremely sensitive, it has poor spatial resolution, and hence, integrated PET scanners are used to co-register PET images with CT or MRI for accurate anatomical localisation. The great advantage of PET as a molecular imaging technique lies in its ability to target specific pathologic features or processes of interest.

^18^F-fluorodeoxyglucose (FDG) is the most commonly used PET tracer, both in clinical practice and research relating to vascular inflammation. As a radiolabelled glucose analogue, ^18^F-FDG is taken up by all metabolically active cells that rely on glucose as a substrate, including macrophages and other inflammatory cells (e.g. neutrophils and lymphocytes). Stemming from the initial proof-of-principle study to evaluate ^18^F-FDG for imaging carotid artery inflammation [4], a surfeit of studies have confirmed the utility of this tracer in atherosclerosis imaging. Indeed, numerous studies have confirmed a strong correlation between ^18^F-FDG uptake and histological markers of macrophage density in atherosclerotic plaques, as well as clinical, biochemical, and gene expression markers related to inflammation. This topic has been comprehensively reviewed elsewhere [5, 6].

However, it is also clear that in certain scenarios, significant contributions to vascular PET signals arise from non-macrophage glucose metabolising cells that may or may not be part of the inflammatory response. Moreover, because of avid physiological uptake of ^18^F-FDG by cardiac myocytes, coronary imaging is precluded in up to 50% of patients with this tracer despite the use of stringent myocardial suppression protocols [7, 8]. For these reasons, alternative PET tracers for imaging vascular inflammation are being actively sought.

## Novel PET Tracers for Imaging Atherosclerosis

In atherosclerosis, the search for PET tracers for imaging inflammation and related pathophysiological processes was initially focused on identifying high-risk or “vulnerable” atherosclerotic plaques. However, given the low positive predictive value of individual vulnerable plaque detection for prediction of future clinical events, the focus has now shifted towards identifying high-risk patients who may have a high burden of inflamed, vulnerable plaques. The histological features of high-risk plaques include a thin fibrous cap; high macrophage density; a large lipid-rich, necrotic, and hypoxic core; “spotty” microcalcification; and neo-angiogenesis. While several non-invasive and invasive imaging techniques are capable of identifying markers associated with histological findings of high-risk plaques, ^18^F-FDG PET is the most widely studied method for detecting plaque inflammation [9].

Novel tracers for atherosclerosis imaging, of which many have been repurposed from oncology imaging, can be broadly categorised into those targeting inflammatory cells and those targeting adjunctive atherosclerotic processes. Some of the most promising approaches for imaging atherosclerosis-related vascular inflammation with PET to date are highlighted below. A more extensive list covering the range of PET tracers tested for vascular inflammation imaging is provided in Table 1.

**Table 1:** Novel tracers for vascular inflammation imaging

Tracer	Molecular target/mechanism	Cellular target	Biological process
Atherosclerosis			
Tracers for imaging inflammatory cells			
^11^C-PK11195 [10] ^18^F-FEDAA1106 [11] ^18^F-FEMPA [12] ^18^F-GE-180 [13]	TSPO	Activated macrophages	Inflammatory cell recruitment/activity
^68^Ga-DOTATATE [14, 18, 19] ^64^Cu-DOTATATE [20, 21] ^68^Ga-DOTANOC [18] ^18^F-FDR-NOC [18] ^68^Ga-DOTATOC [21, 22]	SST_2_ and other somatostatin receptor sub-types	Predominantly “pro-inflammatory” M1 macrophages	‘’
^68^Ga-pentixafor [23–28]	CXCR4	Leukocytes, including monocytes/macrophages and lymphocytes	‘’
^64^Cu-DOTA-DAPTA-comb nanoparticles [30]	CCR5	Monocytes/macrophages	‘’
^64^Cu-DOTA-ECL1i [31, 32]	CCR2	Monocytes/macrophages	‘’
^64^Cu-DOTA-vMIP-II [29] ^64^Cu-vMIP-II-comb nanoparticles [33]	Chemokine receptors (multiple)	Monocytes/macrophages	‘’
^18^F-FOL [34]	Folate receptor β	Macrophages	‘’
^68^Ga-NOTA-MSA [35] ^18^F-FDM [36] ^64^Cu-MMR and ^68^Ga-MMR nanobodies [37]	Mannose receptor	Predominantly “reparative” M2 macrophages	‘’
^18^F-fluorothymidine [38]	Thymidine analogue	Multiple cell types (Cellular proliferation marker)	‘’
^18^F-fluoromethylcholine [39–41] ^11^C-choline [42]	Choline analogues	Multiple cell types (markers of phospholipid metabolism)	‘’
^89^Zr-modified dextran nanoparticles [43], ^18^F-Macroflor [45]	Internalized by phagocytic myeloid cells	Predominately monocytes/macrophages	‘’
Tracers for imaging adjunctive atherosclerotic processes			
^68^Ga-Fucoidan [46] ^64^Cu-DOTA-anti-P-selectin antibodies [47]	P-selectin	Endothelial cells	Endothelial cell activation and margination of circulating monocytes and other inflammatory cells
^18^F-4V [48] ^64^Cu-VCAM nanobody [37]	VCAM-1	Endothelial cells	‘’
^64^Cu-LOX nanobody [37]	LOX-1 receptor for oxidised LDL	Endothelial cells, also macrophages and smooth muscle cells	Uptake of oxidised LDL, also inflammatory cell recruitment/activity
^89^Zr-LA25 [49]	Oxidation-specific epitopes	n/a (by-products of LDL oxidation)	LDL oxidation
^89^Zr-HDL nanoparticles [50]	High-density lipoprotein (HDL)	Macrophages	Cholesterol transport
^18^F-ML-10 [51]	Cell membrane fragments	Multiple cell types	Cellular apoptosis, especially of smooth muscle cells
^18^18F-NaF [8]	Hydroxyapatite	n/a	Microcalcification
^18^F-HX4 [53] ^18^F-FMISO [54, 55] ^62^Cu-ATSM [56]	n/a (hypoxia markers)	n/a	Hypoxia
^18^F-fluciclatide [57] ^18^F-Galacto-RGD [58, 59] ^18^F-Flotegatide [60]	Integrin αvβ3	Activated endothelial cells, also macrophages	Neo-angiogenesis, also inflammatory cell recruitment/activity
^64^Cu-DOTA-C-ANF [61] DOTA-CANF-comb nanoprobe [62]	Natriuretic clearance receptors	Endothelial cells and vascular smooth muscle cells	Neo-angiogenesis
^18^F-florbetaben [63] ^18^F-flutemetamol [64]	Amyloid	n/a	Amyloid within plaque
Large vessel vasculitis			
^11^C-PK11195 [75, 76]	TSPO	Activated macrophages	Granuloma formation, also inflammatory cell recruitment/activity
^68^Ga-DOTATATE [77] ^18^F-FET-βAG-TOCA [77]	SST_2_ receptors	Predominantly “pro-inflammatory” M1 macrophages	‘’

### Tracers for Imaging Inflammatory Cells

Inflammatory cells such as macrophages, which are fundamental to the pathogenesis of atherosclerosis, can be identified by various cell-surface markers or receptors. These markers have been utilised as imaging targets for several classes of PET tracers examined in atherosclerosis.

The translocator protein (TSPO) receptor is situated in the outer mitochondrial membrane and is highly expressed in activated cells of the mononuclear phagocyte lineage. In a study of 32 patients who underwent PET imaging with the TSPO radioligand ^11^C-PK11195, culprit carotid plaques associated with stroke or transient ischaemic attack where highlighted by the tracer [10]. In this study, ^11^C-PK11195 signals were confirmed in macrophage-rich plaque areas when examined histologically [10]. Other TSPO tracers that have potentially lower non-specific binding than ^11^C-PK11195 and are less affected by genetic polymorphisms leading to variable receptor binding affinity are being investigated [11–13].

**Fig. 1:**
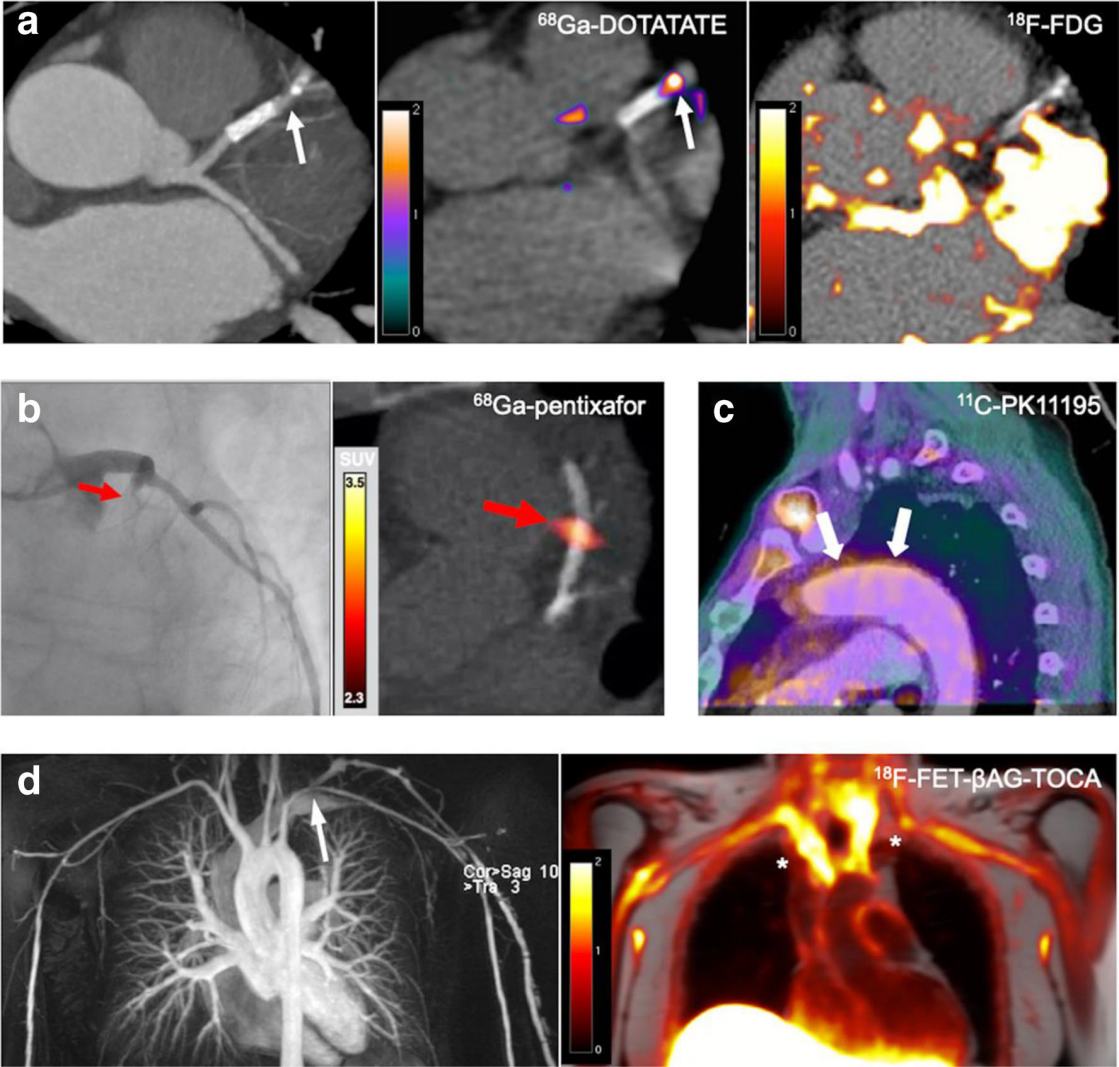
PET imaging of vascular inflammation in atherosclerosis and large-vessel vasculitis. **a** CT coronary angiography (left panel), ^68^Ga-DOTATATE (centre panel), and ^18^F-FDG (right panel). PET/CT imaging in a patient with non-ST segment myocardial infarction due to a culprit left anterior descending artery lesion (arrow). While there is increased ^68^Ga-DOTATATE (SST_2_) PET signal arising from the culprit coronary artery, accurate coronary ^18^F-FDG image interpretation is precluded by diffuse background myocardial tracer uptake. (Reproduced from: Tarkin JM, et al. J Am Coll Cardiol 2017; 69:1774–1791; doi: 10.1016/j.jacc.2017.01.060; Creative Commons user licence https://creativecommons.org/licenses/by/4.0/) [19•]. **b** Coronary angiography (left panel) and ^68^Ga-pentixafor (CXCR4) PET/CT imaging (right panel) showing avid tracer uptake in a culprit left anterior descending artery lesion (arrow) of a patient with acute myocardial infarction. (Reproduced from: Derlin T, et al. J Nucl Med Mol Img 2018; 45:1934–1944; doi.org/10.1007/s00259-018-4076-2; Creative Commons user licence https://creativecommons.org/licenses/by/4.0/) [25•]. **c** ^11^C-PK11195 (TSPO) PET/CT imaging in a patient with active giant cell arteritis showing increased tracer uptake (arrows). (Reproduced from: Pugliese F, et al. J Am Coll Cardiol 2010;56(8):653–61. doi: 10.1016/j.jacc.2010.02.063, with permission from Elsevier) [76]. **d** MR angiography (left panel) showing a chronic left subclavian stenosis (arrow) in a patient with treatment-resistant Takayasu arteritis. In this patient, ^18^F-FET-βAG-TOCA (SST_2_) PET/MRI demonstrates increased tracer signal in the affected vessels (asterisks). (Reproduced from: Tarkin JM, Circ Cardiovasc Imaging 2020;13(6):e010389; doi: 10.1161/CIRCIMAGING.119.010389, with permission from Wolters Kluwer Health Inc.) [77]

Upregulation of somatostatin receptor subtype-2 (SST_2_) occurs in activated macrophages. Initial preclinical studies performed in mice, and retrospective analyses of imaging data from patients who underwent PET imaging as part of oncologic work-up, suggested that the SST_2_ PET ligand ^68^Ga-DOTATATE could be useful for imaging vascular inflammation [14–18]. In a subsequent prospective clinical study of 42 patients with atherosclerosis, ^68^Ga-DOTATATE was found to accurately localise macrophage-related inflammation in atherosclerotic plaques when compared with histological and gene expression analyses. Unlike ^18^F-FDG, low physiological myocardial binding of this tracer permitted reliable analysis of the coronary vasculature [19•](Fig. 1a). Another study, of ^64^Cu-DOTATATE, also found this tracer to be useful for identifying carotid artery inflammation in patients with transient ischaemic attack and identified an association with gene expression of markers in macrophages [20]. Several other clinical somatostatin receptor binding tracers have also been examined for use in atherosclerosis imaging [18, 21, 22].

The C-X-C motif chemokine receptor type 4 (CXCR4) is expressed on the surface of several cell types involved in atherosclerosis, including macrophages and lymphocytes. ^68^Ga-pentixafor binding has been observed in macrophage-rich excised carotid atherosclerotic plaques [23]. Increased arterial ^68^Ga-pentixafor PET signals have also been associated with the presence of clinical cardiovascular risk factors [24]. In a clinical study of 37 patients who underwent ^68^Ga-pentixafor PET after stent-based reperfusion for ST segment elevation myocardial infarction, PET signals were increased in culprit compared with non-culprit lesions [25•] (Fig. 1b). Other studies have also confirmed the utility of this tracer for atherosclerosis imaging [26–28].

PET tracers targeted at C-C chemokine receptor type 5 (CCR5), type 2 (CCR2), and other immune cell chemokine surface receptors have been evaluated in animal models of vascular injury and atherosclerosis [29–32]. In one study, the feasibility of imaging 8 chemokine receptors simultaneously was tested using targeted nanoparticles [33]. Folate receptor β and the mannose receptor are other promising macrophage receptor targets that have been examined in preclinical studies [34–37].

Other approaches for imaging inflammation include PET tracers such as ^18^F-fluorothymidine (FLT), ^18^F-fluoromethylcholine, and ^11^C-choline, which provide markers of cellular proliferation and phospholipid metabolism [38–42]. In one study of 10 consecutive stroke patients scheduled for carotid endarterectomy, ^18^F-fluoromethylcholine uptake was higher in symptomatic carotid arteries than the contralateral asymptomatic side and was significantly correlated with the degree of CD68 macrophage staining in endarterectomy specimens [41].

Radiolabelled nanoparticles, such as modified dextrans, which are directly internalised by myeloid phagocytic cells have also been successfully imaged using PET [43–45].

### Tracers for Imaging Adjunctive Atherosclerotic Processes

Endothelial activation and margination of circulating monocytes and other inflammatory cells occur at an early state in the pathogenesis of atherosclerosis. P-selectin and vascular cell adhesion protein-1 (VCAM-1) are endothelial cell adhesion molecules involved in this process, which have been targeted by experimental PET tracers in preclinical studies [37, 46–48].

PET tracers have also been tested in preclinical atherosclerosis models for imaging cholesterol transport, markers of low-density lipoprotein (LDL) oxidation, and apoptosis, which lead to the formation of lipid-rich necrotic cores [37, 49–51].

Microcalcification is another important feature of atherosclerosis, which is associated with vulnerable plaques. Arterial microcalcification that is below the resolution of a CT scanner can be visualised by ^18^F-NaF, which binds to exposed hydroxyapatite. This tracer has been extensively evaluated for use in atherosclerosis imaging in recent years [52]. In a prospective clinical trial of 80 patients with myocardial infarction or stable angina, ^18^F-NaF was able to better differentiate culprit from non-culprit coronary arterial plaques than ^18^F-FDG and showed increased signals in stable lesions with high-risk plaque features confirmed by intravascular ultrasound [8].

Hypoxia is a feature of the lipid-rich necrotic core within plaques and contributes to angiogenesis in atherosclerosis. In two prospective clinical studies of patients with carotid artery disease who underwent PET imaging with either ^18^F-HX4 or ^18^F-FMISO for measuring hypoxia, tracer uptake was increased in relation to carotid plaques identified by MRI and symptomatic carotid lesions, respectively [53, 54]. In both studies, strong correlations were observed between PET markers of hypoxia and carotid arterial ^18^F-FDG uptake, supporting prior data showing that hypoxia augments ^18^F-FDG uptake in atherosclerotic plaques. Preclinical research performed using ^18^F-FMISO and another hypoxia tracer, ^62^Cu-ATSM, in rabbit models of atherosclerosis is also consistent with these findings in patients [55, 56].

It may also be possible to image neo-angiogenesis relating to high-risk atherosclerotic plaques by targeting integrins, such as αvβ3, with PET ligands. Integrin αvβ3 is expressed by activated endothelial cells, as well as macrophages. In a study of 46 patients with atherosclerosis who underwent PET imaging with the αvβ3 tracer ^18^F-fluciclatide, uptake of this tracer in the aorta was higher in patients with myocardial infarction than stable angina and was correlated with measures of aortic atherosclerotic burden [57]. Other αvβ3-targeted tracers that have been evaluated for use in atherosclerosis include ^18^F-Galacto-RGD and ^18^F-Flotegatide [58–60]. The imaging of natriuretic clearance receptors, which in atherosclerosis are upregulated in endothelial cells as well as vascular smooth muscle cells, has also been tested in preclinical studies [61, 62].

Amyloid ß peptides represent another potential atherosclerosis imaging target, as these peptides exist within human atherosclerotic plaques. Vascular deposition of amyloid ß is associated with inflammatory changes in the vessel wall and microvasculature and could contribute to the known link between cardiovascular disease and Alzheimer’s disease. The feasibility of amyloid PET imaging in carotid arterial atherosclerosis has been demonstrated using ^18^F-florbetaben and ^18^F-flutemetamol [63, 64].

## Novel PET Tracers for Imaging Large-Vessel Vasculitis

LVV comprises a group of chronic, systemic granulomatous diseases, including giant cell arteritis and Takayasu arteritis, which lead to progressive injury of the aorta and its main branches, affecting the organs and limbs supplied. The use of ^18^F-FDG PET to confirm the diagnosis of active LVV in the extracranial vessels is well-established in clinical practice and supported by international guideline recommendations [65–67]. Indeed, a meta-analysis of 298 patients from 9 studies showed that ^18^F-FDG PET demonstrated a pooled sensitivity of 88% and specificity of 81% for diagnosis of LVV [68]. However, it is important to note that the diagnostic accuracy of ^18^F-FDG PET for LVV is significantly reduced after high-dose steroids which are given for more than 3 days [69]. Although the use of ^18^F-FDG for the detection of temporal arteritis in GCA or coronary involvement in Takayasu arteritis is often hampered by background tracer uptake from the brain and myocardium, respectively, a study of 64 patients found ^18^F-FDG PET to be accurate for biopsy-proven temporal arteritis [70]. As well as confirming the diagnosis, ^18^F-FDG PET imaging reveals the distribution of affected arteries and can identify areas of pre-stenotic disease to prompt early intervention.

However, the utility of ^18^F-FDG PET for monitoring therapeutic response is uncertain. Vascular tracer uptake is observed in a high proportion of patients with clinically inactive disease, as demonstrated in an observational study of 56 patients with LVV, where it may represent vessel wall remodelling, atherosclerosis, or another process besides active arteritis [71]. A discordance between clinical disease severity and vascular ^18^F-FDG uptake after 6 months of biologic therapy for LVV was also highlighted in another study [72]. Moreover, in patients with LVV who require vascular surgery, peri-prosthetic graft uptake is commonly observed and appears unrelated to clinical disease activity, C-reactive protein (CRP), or risk of subsequent disease progression [73]. For these reasons, there is a real clinical need for more specifically targeted PET ligands in the management of LVV [74].

As a chronic granulomatous disease, macrophages are a key component of the underlying disease process in LVV. One tracer that has previously been evaluated for imaging macrophage infiltration in LVV is the TSPO ligand ^11^C-PK11195 [75, 76]. In a study of 15 patients with clinically suspected active vasculitis or asymptomatic control subjects, increased ^11^C-PK11195 arterial uptake was observed in patients with active disease [76] (Fig. 1c). Macrophage SST_2_ PET imaging in LVV is the subject of an ongoing clinical study (clinical trials.gov: NCT04071691), for which the initial data appear promising. In the first clinical description of SST_2_ PET/MRI in patients with Takayasu arteritis from this ongoing study, ^68^Ga-DOTATATE and ^18^F-FET-βAG-TOCA accurately identified active arteritis in a patient with relapsing disease and another with treatment-resistant disease [77] (Fig. 1d).

## Targeting Specific Components of the Immune Response

Research into novel PET tracers for imaging vascular inflammation has generated a wealth of encouraging data for a number of these approaches in atherosclerosis and LVV. Ultimately, PET imaging provides the opportunity to identify and characterise vascular lesions by phenotypic markers and processes relating to underlying inflammatory cell activity. For example, certain PET tracers are targeted to individual cell receptors on classically or alternatively activated macrophages known to be important at various stages in atherosclerotic plaque formation, rupture, and healing. However, the true selectively of many of these PET tracers for individual inflammatory cell types remains unknown. While important insights have been gained by studies attempting to answer this question using in vitro cell lines [78], ex vivo histological comparisons, and gene expression data to validate imaging findings, none of these experimental conditions can accurately replicate the in vivo environment. This is an important hurdle for future research in the field, which may be helped by advances in genomic methods such as single cell RNA sequencing and spatial transcriptomics. Indeed, the clinical translational value of novel tracers applied to examine individual components of the human immune response hinges on an ability to accurately understand the molecular and biological basis of PET signals, in order to apply valid clinical interpretation.

## PET Imaging as a Surrogate Marker of Drug Efficacy in Clinical Trials

PET imaging has been applied in numerous clinical trials to examine the effects of cardiovascular therapies on vascular wall inflammation. For example, ^18^F-FDG PET has been used in the evaluation of cholesterol-lowering drugs such as statins and newer therapies that inhibit cholesteryl ester transfer protein, lipoprotein-associated phospholipase A2 (Lp-PLA2), p38 mitogen-activated protein kinase, and proprotein convertase subtilisin-kexin 9 (PCSK9) [79–83]. Overall, the results of these initial PET studies have been consistent with findings of subsequent larger clinical outcome trials [84]. For example, in a randomised placebo-controlled trial, dampening of carotid arterial ^18^F-FDG signals was observed after treatment with a PCSK9 inhibitor that is known to reduce cardiovascular risk through intensive low-density lipoprotein lowering, with a neutral effect on CRP [82]. In another single-arm pilot study, arterial ^18^F-FDG uptake was reduced in individuals with treated human immunodeficiency virus and established cardiovascular disease or risk factors following a single injection of the IL-1β antagonist canakinumab [85]. Moreover, reductions in vascular inflammation have also been observed in clinical trials of patients treated for systemic inflammatory diseases associated with increased cardiovascular risk including diabetes, rheumatoid arthritis, psoriasis, and ankylosing spondylitis [86–89]. The results of observational ^18^F-FDG PET studies involving drug interventions in LVV are discussed above.

Other PET tracers that may provide more precise markers of inflammation than ^18^F-FDG could have an advantage for tracking the effects of cardiovascular therapies longitudinally. ^68^Ga-DOTATATE PET is currently being examined for use as an outcome measure in clinical trials of a PCSK9 inhibitor (clinicaltrials.gov: NCT04073810) and the diabetic medication semaglutide (clinicaltrials.gov: NCT04032197). Taking another approach, in a placebo-controlled study of dual anti-platelet therapy with ticagrelor in patients with multi-vessel coronary disease, ^18^F-NaF PET imaging was used to select participants for inclusion on the basis of avid coronary tracer uptake [90•]. Although a negative study, this work exemplifies a potential role of PET imaging as a means of enriching high-risk patient populations in clinical trials before randomisation.

## PET Imaging for Identification and Stratification of High-Risk Patients

As we sit on the brink of a new era of immune-targeted therapies for cardiovascular disease, PET imaging research can potentially help find new ways to identify patients with residual on-treatment vascular inflammation who would most benefit from newly emerging therapies. The landmark CANTOS [2••] and COLCOT [3••] trials established the proof-of-principle that immunomodulatory therapies can improve clinical outcomes in patients with atherosclerotic cardiovascular disease. Yet, other trials of anti-inflammatory agents, such as the Cardiovascular Inflammation Reduction Trial (CIRT) study of low-dose methotrexate, have not reached the same conclusion [91]. A key difference between these studies is that participants in CANTOS were selected on the basis of an elevated CRP, whereas those enrolled in CIRT had a low CRP. Compared with CRP, which although easy to measure represents a downstream marker of systemic inflammation or infection that is far removed from the affected tissue, PET imaging can provide a more direct measure of inflammation arising in the arterial wall.

In this context, PET imaging could either be used to further stratify select groups of high-risk patients or to help identify novel valid non-PET markers of inflammation. However, although a link between arterial inflammation and future cardiovascular risk identified by ^18^F-FDG PET can be implied through its association with clinical risk factors, serum biomarkers, high-risk plaque features, stroke recurrence, and major adverse clinical events in retrospective analyses of large PET imaging datasets [92–95], definitive prospective clinical outcome data is awaited. Studies linking increased arterial ^18^F-FDG uptake with adverse clinical outcomes in patients with psychological stress and chronic noise exposure provide further evidence that PET markers of inflammation can help identify high-risk patients [96, 97].

The prognostic potential of novel PET markers of inflammation and related pathological processes for predicting future cardiovascular events remains to be determined. A prospective study of 40 patients with symptomatic peripheral arterial disease found that PET imaging with both ^18^F-FDG and ^18^F-NaF was excellent for determining risk of restenosis following percutaneous femoral artery angioplasty [98]. Whether these findings are also applicable to coronary disease has yet to be tested. However, a post hoc analysis of prospective observational data from 293 patients showed that fatal and non-fatal myocardial infarction occurred only in patients with increased baseline coronary microcalcification as assessed by ^18^F-NaF PET imaging during a 42-month follow-up period. In fact, ^18^F-NaF PET imaging outperformed both clinical risk scores and coronary artery calcification scoring on receiver operator curve analysis in this study and was independently associated with a 7-fold increased risk in patients with the highest total coronary ^18^F-NaF activity [99•]. The ability of coronary ^18^F-NaF PET to predict recurrent events in patients with recent myocardial infarction and multi-vessel coronary disease is the subject of an ongoing prospective multi-centre clinical trial (clinicaltrials.gov: NCT02278211).

## Conclusions

Current uses of PET for imaging vascular inflammation in the clinic are limited. However, in the future, an ability to interrogate key components of vascular immune responses and systemic inflammatory pathways using PET with ^18^F-FDG or novel tracers could help further the understanding of underlying disease mechanisms in atherosclerosis and vasculitis and inform the design or use of newly emerging immunomodulatory therapies in high-risk patient populations. Research efforts should continue if these roles are to be realised.
